# Exosomes, the message transporters in vascular calcification

**DOI:** 10.1111/jcmm.13692

**Published:** 2018-06-12

**Authors:** Chao Zhang, Kun Zhang, Feifei Huang, Weijing Feng, Jie Chen, Huanji Zhang, Jingfeng Wang, Pei Luo, Hui Huang

**Affiliations:** ^1^ Guangdong Provincial Key Laboratory of Malignant Tumor Epigenetics and Gene Regulation Department of Cardiology Sun Yat‐sen Memorial Hospital Sun Yat‐sen University Guangzhou China; ^2^ RNA Biomedical Institute Sun Yat‐sen Memorial Hospital Sun Yat‐sen University GuangZhou China; ^3^ Department of Radiation Oncology Sun Yat‐sen Memorial Hospital of Sun Yat‐sen University Guangzhou China; ^4^ Cardiovascular Department The Eighth Affiliated Hospital Sun Yat‐sen University Shenzhen China; ^5^ State Key Laboratories for Quality Research in Chinese Medicines Macau University of Science and Technology Macau China

**Keywords:** exosomes, microRNA, osteogenic phenotype transition, vascular calcification

## Abstract

Vascular calcification (VC) is caused by hydroxyapatite deposition in the intimal and medial layers of the vascular wall, leading to severe cardiovascular events in patients with hypertension, chronic kidney disease and diabetes mellitus. VC occurrences involve complicated mechanism networks, such as matrix vesicles or exosomes production, osteogenic differentiation, reduced cell viability, aging and so on. However, with present therapeutic methods targeting at VC ineffectively, novel targets for VC treatment are demanded. Exosomes are proven to participate in VC and function as initializers for mineral deposition. Secreted exosomes loaded with microRNAs are also demonstrated to modulate VC procession in recipient vascular smooth muscle cells. In this review, we targeted at the roles of exosomes during VC, especially at their effects on transporting biological information among cells. Moreover, we will discuss the potential mechanisms of exosomes in VC.

## 1 INTRODUCTION

Vascular calcification (VC) is attributed to calcium and phosphate (Pi) metabolic dysfunction, osteogenic differentiation, inflammation and so on, leading to major adverse cardiovascular events (MACEs), especially in patients with chronic kidney disease (CKD).^1^, ^2^, ^3^ Furthermore, VC occurs in the intimal and medial layers of vessel wall, which is linked to atherosclerotic plaque burden and consequent rupture.^4^ In some clinical trials, moderate or severe calcification contributes to more MACE occurrences in patients treated with revascularization therapy, compared with non/mild calcification.^5^ As VC increases MACE occurrences, many treatments are designed to counteract with VC, such as statins, Pi binders and so on. However, with more concentrations driving into this field, more shortages of such treatments are presented in the front. A meta‐analysis revealed that statins failed to ameliorate coronary artery calcification procession despite reducing LDL‐c level.^6^ Moreover, Pi binders also enhance VC, which are mediated by calcium contained in such binders.^7^, ^8^ Pi binders are also demonstrated to limit bioavailability of vitamin K2, which further inhibits the activity of mineral deposition factor matrix Gla protein (MGP) to enhance VC occurrence.^9^ MGP is an inhibitory factor for VC and inactivated MGP results in exacerbating VC.^9^ Due to the limitation of present treatments in VC, novel targets and therapies for VC are demanded.^10^, ^11^

Importantly, exosomes have been demonstrated to be involved in VC recently.^11^, ^12^ Exosomes have up‐regulated secretion from vascular smooth muscle cells (VSMCs) in vivo after pro‐calcifying stimulation and become “calcifying” exosomes to induce VC.^11^ Calcium binds with Pi to form hydroxyapatite nodes on the inner and outside of “calcifying” exosomes membranes, which further initializes mineral deposition.^11^ Although these studies did reveal that exosomes participated in the calcification procession through promoting mineral deposition sites formation, they did not discuss exosomes functioning as mediators for RNAs transportation, which is vital for exosome function.^13^

Exosomes are secreted by diverse cells to mediate cell‐to‐cell communications.^14^ However, how exosomes regulating VC is only preliminarily explored recently. It is found that exosomes with diverse origins mainly mediate microRNAs (miRs) transporting to VSMCs in coronary artery calcification.^15^ A bioinformatics analysis revealed that cultured in osteogenic medium, mesenchymal stem cells secreted exosomes with alterations of miRs, comparing with normal culturing.^16^ Such alterations were suggested to accelerate calcification in other mesenchymal stem cells to modulate osteogenic phenotype transition.^16^ Thus, it implies that besides heterogeneous mineral deposition inside vessel wall,^11^ exosomes can also promote VC by transporting messages among cells. In this review, we will summarize the roles of exosomes in VC and analyse the potential mechanisms associated with exosomes in VC.

## 2 EXOSOMES PARTICIPATE IN VC

### 2.1 Biological characters of exosomes

Widely found in body fluid, exosomes represent a group of extracellular vesicles (EVs) with intracellular contents, such as proteins and RNAs, which are transported among cells to mediate cell‐to‐cell communications under certain situations.^17^ Exosomes originate from multivesicular bodies (MVBs) and are loaded with intracellular components upon biogenesis.^17^ It is reported that after shear stress stimulation, EVs secreted from endothelial cells enriched with miR‐143/145 and control the phenotype of VSMCs.^18^ Such miRs transportation via exosomes regulates de‐differentiation of VSMCs, which initializes phenotype transition during VC.^13^, ^18^ Recent study also revealed that increasing exosomes secreted by VSMCs promote VC via mineral deposition.^12^ Attributing to EVs congestion in vascular wall, the calcification spheres contribute to heterogeneity of microcalcification formation via mineral deposition.^19^, ^20^ In the process of mineral deposition, comparisons based on previous researches demonstrated that tiny differences existed between exosomes and matrix vesicles (MVs) in size, morphology and lipid/protein contents, indicating that exosomes share characteristics with MVs during calcification procession.^21^ Such vesicles secreted by VSMCs expressing exosome biomarker CD63 are regarded as exosomes.^11^

**Figure 1:**
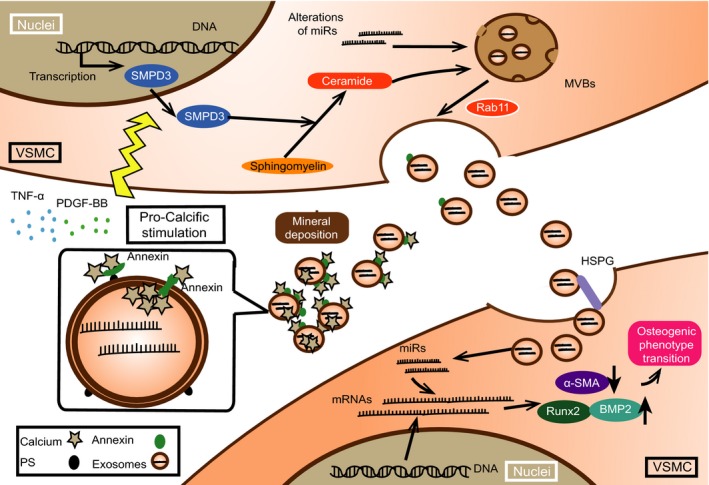
The functions of exosomes during vascular calcification (VC) as initializers and transporters for microRNAs (miRs). Exosomes function as mineral nucleation sites extracellularly and transport miRs among cells targeting at mRNAs in the recipient vascular smooth muscle cells. Exosomes intake further promotes miRs transportation among cells, which is in a heparin sulphate proteoglycans (HSPG)‐dependent manner. Moreover, under pro‐calcific milieu, exosomes secretion is enhanced by sphingomyelin phosphodiesterase 3 (SMPD3)

Moreover, as Clotilde Thery et al suggested, exosomes represent the mixed population of small EVs which transport information among cells as the primary function.^17^ Recently, exosomes derived from calcified VSMCs were proven to enhance calcification in the recipient VSMCs via activating mitogen‐activated protein kinase.^22^ Exosomes are also suggested to deliver intracellular contents such as proteins and RNAs, functioning as message transporters to promote VC.^23^ Emerging evidences revealed that exosomal miRs were significant in diagnosis, prognosis or even therapeutic target selection in patients with cancer and heart failure.^24^, ^25^ Selective enrichments of miRs in exosomes were due to the alterations in parental or donor cells, from which exosomes are secreted or originated.^26^ Of note, exosomes take part in cellular behaviour changes, such as phenotype transition and inflammatory reactions via transporting miRs to interfere with several signalling pathways.^27^, ^28^ Uptake of exosomes by osteoblasts is accelerated by increased receptors expressed on the cell surface, with transporting miRs from osteoclasts under osteoclastogenesis stimulation.^29^ All of secretion, congestion and uptake processions of exosomes modulate VC from different aspects. Thus, exosomes participate in the procession of VC via partially promoting mineral deposition sites formation and transporting miRs as information among cells (Figure 1).

### 2.2 Initializing mineral deposition

Resembling to bone formation, mineral deposition is the characteristic feature of VC and MVs are regarded as the major players of calcification procession.^30^ Elevated calcium combines with Pi to form mineral deposition sites which determines the outcome of calcification.^31^ It is reported that MVs derived from macrophages enhanced ectopic mineralization after culturing in the high calcium/Pi medium.^32^ During VC, MVs are proven to participate in mineral nucleation sites formation, and decreased MVs secretion results in amelioration of VC.^33^, ^34^ It is proven that exosomes, as MVs, obtain mineral compounds to maintain the intracellular mineral metabolism homeostasis, which further aggravates the mineral deposition sites formation.^11^, ^12^

It is reported that exosomes secretion pathway is activated during VC and modulations on such procession exert as novel targets for VC prevention.^11^ Specifically, elevated Pi and calcium and cytokines, including tumour necrosis factor α (TNF‐α) and platelet‐derived growth factor‐BB (PDGF‐BB) enhance exosomes secretion via elevating sphingomyelin phosphodiesterase 3 (SMPD3, also known as neutral sphingomyelinase 2, nSMase2) expression.^11^ Pro‐calcifying stimulation increases the expression of SMPD3/nSMase2 of VSMCs and leads to enhanced calcification.^2^ SMPD3/nSMase2 converts sphingomyelin to ceramide which induces the conjunction of clathrin‐coated microdomains and further promote exosomes secretion.^35^ As phenotype transiton of VC involving cytoskeleton remodelling, such intracellular alterations would promote exosomes secretion via ceramide.^2^, ^36^, ^37^

Moreover, the “calcifying” exosomes secreted during VC are characterized with low MGP contents and high level of hydroxyapatite, which initialize mineral deposition as microcalcifiction.^11^ It is known that Gla‐rich proteins, including MGP, inhibit the nucleation sites formation on the surface of exosomes via binding with externalized phosphatidylserine (PS).^12^ Such inhibitions of mineral‐binding abilities further block calcium deposition in an exosomes‐dependent manner and ameliorate calcification procession.^11^, ^12^ Besides mineral contents inside exosomes, externalized PS combines with calcium‐binding protein (such as Annexin A2, A5, A6), which forms hydroxyapatite deposition inner and outside exosomes.^38^ Moreover, Annexins are loaded into exosomes before releasing.^39^ Extracellular Pi concentration is further enhanced by phosphatases on MVs surface via converting pyrophosphate to provide ectogenic Pi. Choline kinase mutant also enhances some phosphatase activities as compensatory mechanism to accelerate Pi production.^40^ Thus, such evidences indicated that exosomes participated in VC via forming calcium deposition sites, which are attributed to exosomes contents and calcium‐binding abilities.

### 2.3 Transporting miRs to modulate VC

Cell‐to‐cell communication is a key mechanism for VC occurrence.^41^ Recent findings showed that exosomes played important roles in transporting information among cells.^41^ As plenty of works had focused on the roles of exosomes in mineral deposition during VC, limited insights into VC do not clearly explain the exact procession of exosomes as information transporters.^11^ Exosomes mediate information transportation among cells, which are reported to depend on heparin sulphate proteoglycans (HSPG) for the internalization in cancer cells.^42^ However, HSPG protects VSMCs from various toxic substances and circulating inflammatory cells to prevent VC.^43^ Reduced HSPG expression in the extracellular matrix (ECM) exposes HSPG on cell surface, which further mediates bone morphogenetic protein 2 (BMP2) internalization to enhance osteogenic phenotype transition in myoblast cells.^44^ Inhibition of HSPG expression on the cell surface leads to decreased efficiency of exosomes uptake.^29^, ^45^

Functioning as carriers to transport cargos among cells, exosomes trigger some reactions in recipient cells. Exosomes cargos contain RNAs (including mRNAs and miRs), cytokines, lipids and so on.^2^ Exosomes released from mineralizing pre‐osteoblast MC3T3‐b1 cells promote osteogenic differentiation in ST2 cells, which is mediated by the complicated networks formed by exosomal miRs.^46^ Other research also revealed that miRs expression in MVs during VC, suggesting that exosomes might transport vital information during VC.^47^ Despite enhancing the exosomes secretion in VC, elevated SMPD3/nSMase2 expression also modulates miRs sorting into exosomes, and quantitative analysis revealed that inhibition of SMPD3/nSMase2 led to significantly decreased expression of several miRs in exosomes.^48^ Alteration of miRs inside exosomes regulates osteogenic differentiation of human bone‐marrow‐derived mesenchymal stem cells.^16^ Furthermore, these alterations of miRs inside exosomes could augment osteogenic phenotype transition via elevating runt‐related transcription factor 2 (Runx2) expressions and activating several signalling pathways such as Wnt/β‐catenin.^46^

Osteogenic phenotype transition represents as a crucial characteristic of VC, with switching from contractile phenotype to osteoblast‐like cells.^49^ And such procession is mirrored by expression of osteogenic transcription factors such as Runx2 and loss of contractile phenotype such as α‐smooth muscle actin (α‐SMA).^50^ It is revealed that some miRs with elevated expression during VC promote osteogenesis via targeting at anti‐calcification proteins or contractile markers, whereas some other miRs with decreased expression suppress osteogenesis of VSMCs through targeting at osteogenic transcription factors^13^, ^51^, ^52^, ^53^, ^54^, ^55^, ^56^, ^57^, ^58^, ^59^, ^60^, ^61^ (shown in Table 1). As described, some of such miRs, including miR‐133b,^55^ miR‐204,^57^ miR‐211,^55^ alter during VC and are also proven to be transported by exosomes to modulate the biological behaviour in various kinds of recipient cells.^62^, ^63^, ^64^ Such results indicated that exosomes could participate in VC through transporting miRs to influence phenotype transition. However, Ulbing et al^65^ reported that circulating miR‐223 was down‐regulated in CKD patients and decreased expression of miR‐223 was regarded as a risk factor for VC occurrence which might be packaged into exosomes. Moreover, miR‐223 expression is up‐regulated in VSMCs under elevated Pi stimulation.^56^ Such contradiction implies that besides message transporters, exosomes function more than what the present knowledge obtains and more attentions need to be paid in such field.

**Table 1:** Targets and expression changes of different miRs in VC procession

miR(s)	Target molecule	Pro‐calcific stimulation	Cell Source/Tissue	Function	Reference number	miRNA expression
miR‐29b	ACVR2A CTNNBIP	Pi‐induced	Rat VSMCs	Inhibition of osteoblast differentiation	55	↓
miR‐30b/c	Runx2	rhBMP2‐induced	Human coronary artery SMCs	Inhibition of osteoblast differentiation	51	↓
miR‐32	PTEN	β‐glycerophosphate‐induced	Mouse VMSCs	Promotion of osteoblast differentiation	52	↑
miR‐34b/c	SATB2	Aldosterone‐induced	Rat VSMCs	Suppression of osteogenesis transdifferentiation	53	↓
miR‐125b	Osterix	β‐glycerophosphate‐induced	Human coronary artery SMCs	Decreasing ALP expression and matrix mineralization	54	↓
miR‐133b	Runx2	Pi‐induced	Rat VSMCs	Inhibition of osteoblast differentiation	55	↓
miR‐143/145	KLF4/KLF5	Pi‐induced	HAVSMCs	Phenotype transition preservation	56	↓
miR‐155	AT1R	CKD(transgenic rat)	Rat VSMCs	Inhibitions to VC	13	↓
miR‐204	Runx2	β‐glycerophosphate‐induced	Mouse VSMCs	Inhibition of osteoblast differentiation	57	↓
miR‐211	Runx2	Pi‐induced	Rat VSMCs	Inhibition of osteoblast differentiation	55	↓
miR‐223	Mef2c/RhoB	Pi‐induced	Human VSMCs	Phenotype transition from contractile to synthesis and calcification induction	56	↑
miR‐712	NCKX4	Klotho homozygous mutant	Mouse VSMCs	Disrupt calcium transporters and promote calcium deposition	60	↑
miR‐714	PMCA1	Klotho homozygous mutant	Mouse VSMCs	Disrupt calcium transporters and promote calcium deposition	60	↑
miR‐762	NCX1	Klotho homozygous mutant	Mouse VSMCs	Disrupt calcium transporters and promote calcium deposition	60	↑
miR‐2861	HDAC5	β‐glycerophosphate‐induced	Mouse VMSCs	Promotion of osteoblast differentiation	61	↑
miR‐3960	HoxA2	β‐glycerophosphate‐induced	Mouse VMSCs	Increasing osteoblastogenesis	61	↑

ACVR2A, activin A receptor type II A; AT1R, angiotensin type 1 receptor; CTNNBIP, β‐catenin interacting protein; HDAC5, histone deacetylase 5; HoxA2, homeobox A2; Mef2C, myocyte enhancer factor 2C; NCKX4, sodium/calcium exchange member 1; NCX1, sodium/calcium exchange member 1; PMCA1, plasma membrane calcium pump isoform 1; PTEN, phosphate and tensin homologue; RhoB, ras homologue family member B; SATB2, special AT‐rich sequence‐binding protein 2.

## 3 POTENTIAL REGULATORY MECHANISMS OF EXOSOMES IN VC

### 3.1 Autophagy

Autophagy is aimed to digest intracellular proteins and organelles when cells encounter with emergent situations, such as stress responses.^66^ A series of studies have focused on the relationship between autophagy and VC, and it seems that autophagy ameliorates such procession through AMP‐activated protein kinase (AMPK) activation under Pi‐induced situation.^67^ Autophagy involves autophagosomes formation mediated by LC3, Beclin1 and autophagic flux activated by autophagosomes fusing with lysosomes.^68^ Up‐regulating LC3 and Beclin1 expression blocks calcium deposition in high Pi stimulation, which indicates that autophagy might have the inhibitory role in VC.^67^ It is also reported that 7‐ketecholesterol, a VC inducer, promotes VC through lysosomes dysfunction which blocks the fusion of autophagosomes and lysosomes.^69^

Recently, autophagy is believed to be enhanced by high Pi stimulation and suppresses MVs secretion which further forms mineral nucleation sites and consequently ameliorates calcification in VSMCs.^34^ It is well documented that autophagy accelerates MVBs degradation and decreases exosomes secretion, which is mediated by autophagosome‐lysosome fusion.^21^ Briefly, MVBs move to neighbourhood of the cell membrane and then dock to the membrane for exosomes releasing, which is regulated by Rab GTPases.^70^ One of such GTPase, Rab11, then induces exosomes secretion by promoting MVBs docking and fusing to cytomembrane in a calcium‐dependent manner.^71^ Rab11 also enhances autophagosomes fusing with MVBs to form amphisomes under interferon‐γ treatment, which promotes Annexins loading into exosomes.^39^

Moreover, autophagy seems to interfere with miRs loading into exosomes during VC. It is reported that heterogeneous ribonuclear protein A2/B1 (hnRNPA2/B1) plays a vital role in promoting miRs loading into exosomes.^72^ It is believed that small ubiquitin‐like modifier (SUMOylation) of hnRNPA2/B1 promotes miRs loading into exosomes.^72^ In addition, Ubc9, the E2‐conjugating enzymes mediating SUMOylation, is degraded in autophagy procession.^73^ Thus, autophagic flux partially decreases exosomes secretion and miRs loading into exosomes, which might interfere with mineral deposition and osteogenic phenotype transition. However, more researches need to distinguish the exact function of autophagy in VC.

### 3.2 Inflammation

It has been known that inflammation promotes VC, which is modulated by inflammatory cytokines secreted from inflammatory cells, such as macrophages.^74^ Expression of TNF‐α and interleukin (IL) family members, such as IL‐1β and IL‐6, is increased and such cytokines play pivotal roles in the procession of VC.^75^ These cytokines enhanced the expression of BMP2 and reduced MGP expression, further promoting VC procession in VSMCs.^76^ It is also reported that exosomes collected from body fluid promote inflammation.^77^ Previous report indicates that ceramide is elevated due to inflammatory stimulation and promotes VC.^78^

Moreover, macrophages are involved in inflammatory reaction during VC. Derived from monocytes, macrophages are recruited and activated in the calcification area to initialize the mineral deposition, which further enhances the production of inflammatory cytokines.^79^ It is reported that in metabolic disorders, exosomes derived from macrophages shuttle miR‐155 among cells to modulate insulin sensitivities in insulin target recipient cells.^80^ MiRs‐223 is also proven to be transferred by microvesicles from macrophages, and such microvesicles include exosomes and other kinds of EVs.^81^ Both miR‐155 and miR‐223 are also proven to modulate VC,^13^, ^56^ suggesting that besides promoting inflammatory cytokines secretion, macrophages participate in VC via an exosomal miRs‐dependent manner.

In addition, transforming growth factor β (TGF‐β) signalling pathway is proven to promote VC, which is related to inflammation.^82^ High Pi induces activation of TGF‐β/Smad2/3 in VSMCs and Smads modulate specific genes transcription, including SMPD3/nSMase2 which converts sphingomyelin to ceramide.^48^, ^83^, ^84^ However, miR‐29b is proven to inhibit TGF‐β/Smad3 axis activation via targeting at Smad3 and alteration of exosomal miR‐29b modulates such mRNAs expression in the recipient infected cells concerning HIV study.^85^, ^86^ All these results indicate that exosomes modulate inflammation via mediating miRs transportation during VC.

### 3.3 Oxidative stress

Intracellular calcium overloading triggers disruption of superoxide metabolism, and further induces oxidative stress. Excessive production of reactive oxygen species (ROS) promotes VC via inducing osteogenic phenotype transition.^87^ Advanced glycation end‐products (AGEs) are the key factors for ROS production in diabetes mellitus (DM) patients, which activates the receptors to initialize oxidative stress procession. It was found that in DM, AGE up‐regulated ROS production, elevated alkaline phosphatase (ALP) activity and promoted VC via receptors for advanced glycation end‐products (RAGEs).^88^ In another study, Kay et al^89^ demonstrated that AGE/RAGE axis accelerated ROS production via nicotinamide adenine dinucleotide phosphate oxidase 1 (Nox1) to enhance oxidative stress in VSMCs and subsequently enhanced VC. It has been shown that exosomes are associated with oxidative stress. Patel et al^90^ previously found that exosomes from breast cancer cells promoted ROS production in the recipient primary mammary epithelial cells. It is also reported that miR‐30 was down‐regulated after calcification stimulation, which also targeted at RAGEs to modulate AGE/RAGE activity and further decreased oxidative stress level.^51^, ^91^ In fact, miR‐30 could be packed into exosomes and transport information among endothelial cells and mesenchymal stem cells.^92^ The expression of miR‐210 is also proven to be decreased in VC,^52^ and exosomal miR‐210 also ameliorated ROS production in the recipient endothelial cells.^93^ Such results indicated that exosomes could regulate the oxidative stress via modulating ROS production.

### 3.4 Immune response

Immune response is composed of innate and adaptive immunity, which is recently regarded as a major player in the occurrence of cardiovascular disease.^94^ Regulatory T (Treg) cells are of great significance in immune response, which might negatively regulate inflammatory reaction.^94^ In haemodialysis patients, coronary artery calcification score is negatively correlated with Treg cell frequencies and Treg/T‐helper cell 17 functional disequilibrium is also vital in such procession.^95^, ^96^ Exosomes are proven to be involved in Treg cells modulation. Exosomes derived from Treg cells transport exosomal contents including miRs to the recipient conventional T cells or recipient cells in tumour tissue, further modulating immune response or intracellular translation procession.^97^ Indeed, Treg cell is regarded as the suppressive effector in immune system by delivering miRs via exosomes.^98^ It is reported that Treg cells transfer miR‐155 to recipient conventional T cells.^99^ Importantly, miR‐155 is vital in VC procession,^13^ and exosomal miR‐155 derived from Treg cells might function as an additional source of miRs during VC. Thus, exosomes might be a novel interaction point between immune response and VC procession, and such interaction may depend on the miRs transportation.

### 3.5 Other mechanism relating to exosomes during VC

Besides the mechanisms described above, mechanical stretch is regarded as a potential novel player of VC. Exosomes may regulate VC procession through this mechanism. Mechanical environment is recently proven to participate in calcification procession. Balachandran et al^100^ reported that cyclic mechanical stretch could promote aortic valve calcification via elevating Runx2 expression and ALP activity. Mechanical membrane stretch enhanced exosomes secretion in cardiomyocytes, and contents inside exosomes were altered due to the mechanical environment.^101^ Moreover, it was reported that under shear stress stimulation, BMP4 expression was down‐regulated in endothelial cells, which is vital for osteogenic transition during VC.^102^ Also, shear stress promotes miR‐143 loading into exosomes rather than other miRs in endothelial cells, indicating that mechanic environment has effect on selective miRs secretion via exosomes.^103^ Thus, mechanical environment is vital in the procession of VC via alterations of miRs inside exosomes and exosomes secretion.

## 4 CONCLUSION

**Figure 2:**
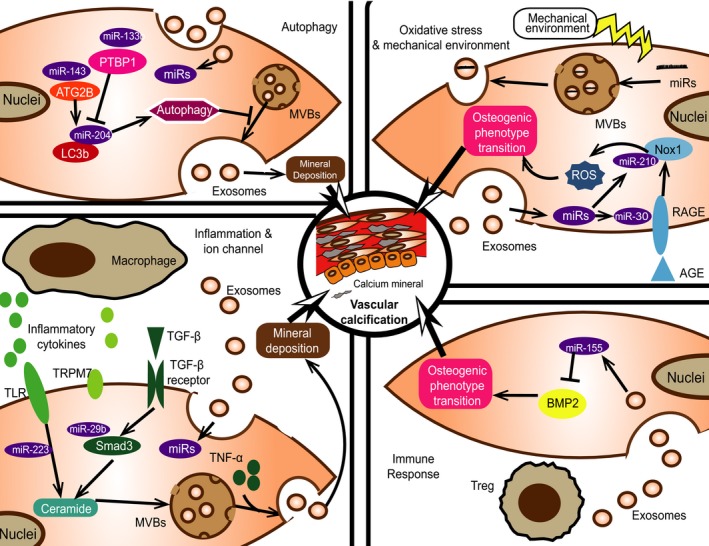
The potential regulatory mechanisms of exosomes during vascular calcification (VC). Several mechanisms of VC occurrence are modulated by exosomes, including autophagy, inflammation, oxidative stress and immune response. Transporting different miRs among cells, exosomes modulate several signalling pathways and further interfere with VC

Vascular calcification elevates the probabilities for patients to encounter with MACEs. In this review, we have discussed the roles of exosomes as message transporters in VC. Exosomes accelerate VC through mediating miRs transportation among cells to regulate autophagy, inflammation, oxidative stress, immune response and other possible mechanisms (Figure 2). Interfering exosomes secretion and miRs alterations inside might provide novel targets for treating VC.

## CONFLICT OF INTEREST STATEMENT

The authors declare that they have no conflict of interest.

## References

[1] Geisel MH , Bauer M , Hennig F , et al. Comparison of coronary artery calcification, carotid intima‐media thickness and ankle‐brachial index for predicting 10‐year incident cardiovascular events in the general population. Eur Heart J. 2017;38:1815‐1822. 28379333 10.1093/eurheartj/ehx120

[2] Kapustin AN , Shanahan CM . Emerging roles for vascular smooth muscle cell exosomes in calcification and coagulation. J Physiol. 2016;594:2905‐2914. 26864864 10.1113/JP271340 PMC4887700

[3] Zhang K , Zhang Y , Feng W , et al. Interleukin‐18 enhances vascular calcification and osteogenic differentiation of vascular smooth muscle cells through Trpm7 activation. Arterioscler Thromb Vasc Biol. 2017;37:1933‐1943. 28860220 10.1161/ATVBAHA.117.309161

[4] Wu M , Rementer C , Giachelli CM . Vascular calcification: an update on mechanisms and challenges in treatment. Calcif Tissue Int. 2013;93:365‐373. 23456027 10.1007/s00223-013-9712-z PMC3714357

[5] Genereux P , Madhavan MV , Mintz GS , et al. Ischemic outcomes after coronary intervention of calcified vessels in acute coronary syndromes. pooled analysis from the horizons‐ami (harmonizing outcomes with revascularization and stents in acute myocardial infarction) and acuity (acute catheterization and urgent intervention triage strategy) trials. J Am Coll Cardiol. 2014;63:1845‐1854. 24561145 10.1016/j.jacc.2014.01.034

[6] Henein MY , Owen A . Statins moderate coronary stenoses but not coronary calcification: results from meta‐analyses. Int J Cardiol. 2011;153:31‐35. 20843566 10.1016/j.ijcard.2010.08.031

[7] Block GA , Wheeler DC , Persky MS , et al. Effects of phosphate binders in moderate Ckd. J Am Soc Nephrol. 2012;23:1407‐1415. 22822075 10.1681/ASN.2012030223 PMC3402292

[8] Drueke TB , Massy ZA . Phosphate binders in Ckd: bad news or good news? J Am Soc Nephrol. 2012;23:1277‐1280. 22797178 10.1681/ASN.2012060569

[9] Neradova A , Schumacher SP , Hubeek I , Lux P , Schurgers LJ , Vervloet MG . Phosphate binders affect vitamin K concentration by undesired binding, an in vitro study. BMC Nephrol. 2017;18:149. 28464802 10.1186/s12882-017-0560-3 PMC5414218

[10] Siltari A , Vapaatalo H . Vascular calcification, vitamin K and warfarin therapy ‐ possible or plausible connection? Basic Clin Pharmacol Toxicol. 2017;122:19‐24. 28639365 10.1111/bcpt.12834

[11] Kapustin AN , Chatrou ML , Drozdov I , et al. Vascular smooth muscle cell calcification is mediated by regulated exosome secretion. Circ Res. 2015;116:1312‐1323. 25711438 10.1161/CIRCRESAHA.116.305012

[12] Kapustin AN , Schoppet M , Schurgers LJ , et al. Prothrombin loading of vascular smooth muscle cell‐derived exosomes regulates coagulation and calcification. Arterioscler Thromb Vasc Biol. 2017;37:e22‐e32. 28104608 10.1161/ATVBAHA.116.308886

[13] Chen NX , Kiattisunthorn K , O'Neill KD , et al. Decreased microrna is involved in the vascular remodeling abnormalities in chronic kidney disease (Ckd). PLoS ONE. 2013;8:e64558. 23717629 10.1371/journal.pone.0064558 PMC3661525

[14] Raposo G , Stoorvogel W . Extracellular vesicles: exosomes, microvesicles, and friends. J Cell Biol. 2013;200:373‐383. 23420871 10.1083/jcb.201211138 PMC3575529

[15] Boulanger CM , Loyer X , Rautou PE , Amabile N . Extracellular vesicles in coronary artery disease. Nat Rev Cardiol. 2017;14:259‐272. 28150804 10.1038/nrcardio.2017.7

[16] Xu JF , Yang GH , Pan XH , et al. Altered microrna expression profile in exosomes during osteogenic differentiation of human bone marrow‐derived mesenchymal stem cells. PLoS ONE. 2014;9:e114627. 25503309 10.1371/journal.pone.0114627 PMC4263734

[17] Tkach M , Thery C . Communication by extracellular vesicles: where we are and where we need to go. Cell. 2016;164:1226‐1232. 26967288 10.1016/j.cell.2016.01.043

[18] Hergenreider E , Heydt S , Treguer K , et al. Atheroprotective communication between endothelial cells and smooth muscle cells through mirnas. Nat Cell Biol. 2012;14:249‐256. 22327366 10.1038/ncb2441

[19] Hutcheson JD , Goettsch C , Bertazzo S , et al. Genesis and growth of extracellular‐vesicle‐derived microcalcification in atherosclerotic plaques. Nat Mater. 2016;15:335‐343. 26752654 10.1038/nmat4519 PMC4767675

[20] Otsuka F , Sakakura K , Yahagi K , Joner M , Virmani R . Has our understanding of calcification in human coronary atherosclerosis progressed? Arterioscler Thromb Vasc Biol. 2014;34:724‐736. 24558104 10.1161/ATVBAHA.113.302642 PMC4095985

[21] Shapiro IM , Landis WJ , Risbud MV . Matrix vesicles: are they anchored exosomes? Bone. 2015;79:29‐36. 25980744 10.1016/j.bone.2015.05.013 PMC4501874

[22] Chen NX , O'Neill KD , Moe SM . Matrix vesicles induce calcification of recipient vascular smooth muscle cells through multiple signaling pathways. Kidney Int. 2017;93:343‐354. 29032812 10.1016/j.kint.2017.07.019 PMC8211355

[23] Tintut Y , Demer LL . Exosomes: nanosized cellular messages. Circ Res. 2015;116:1281‐1283. 25858057 10.1161/CIRCRESAHA.115.306324 PMC5127443

[24] Huang X , Liang M , Dittmar R , Wang L . Extracellular micrornas in urologic malignancies: chances and challenges. Int J Mol Sci. 2013;14:14785‐14799. 23863690 10.3390/ijms140714785 PMC3742273

[25] Yang VK , Loughran KA , Meola DM , et al. Circulating exosome microrna associated with heart failure secondary to myxomatous mitral valve disease in a naturally occurring canine model. J Extracell Vesicles. 2017;6:1350088. 28804599 10.1080/20013078.2017.1350088 PMC5533140

[26] Skog J , Wurdinger T , van Rijn S , et al. Glioblastoma microvesicles transport RNA and proteins that promote tumour growth and provide diagnostic biomarkers. Nat Cell Biol. 2008;10:1470‐1476. 19011622 10.1038/ncb1800 PMC3423894

[27] Pinto S , Cunha C , Barbosa M , Vaz AR , Brites D . Exosomes from Nsc‐34 cells transfected with Hsod1‐G93a are enriched in Mir‐124 and drive alterations in microglia phenotype. Front Neurosci. 2017;11:273. 28567000 10.3389/fnins.2017.00273 PMC5434170

[28] Dalvi P , Sun B , Tang N , Pulliam L . Immune activated monocyte exosomes alter micrornas in brain endothelial cells and initiate an inflammatory response through the Tlr4/Myd88 pathway. Sci Rep. 2017;7:9954. 28855621 10.1038/s41598-017-10449-0 PMC5577170

[29] Sun W , Zhao C , Li Y , et al. Osteoclast‐derived microrna‐containing exosomes selectively inhibit osteoblast activity. Cell Discov. 2016;2:16015. 27462462 10.1038/celldisc.2016.15 PMC4886818

[30] Doherty TM , Asotra K , Fitzpatrick LA , et al. Calcification in atherosclerosis: bone biology and chronic inflammation at the arterial crossroads. Proc Natl Acad Sci U S A. 2003;100:11201‐11206. 14500910 10.1073/pnas.1932554100 PMC208734

[31] Yang H , Curinga G , Giachelli CM . Elevated extracellular calcium levels induce smooth muscle cell matrix mineralization in vitro. Kidney Int. 2004;66:2293‐2299. 15569318 10.1111/j.1523-1755.2004.66015.x

[32] Chen Q , Bei JJ , Liu C , et al. Hmgb1 induces secretion of matrix vesicles by macrophages to enhance ectopic mineralization. PLoS ONE. 2016;11:e0156686. 27243975 10.1371/journal.pone.0156686 PMC4887028

[33] Kapustin AN , Davies JD , Reynolds JL , et al. Calcium regulates key components of vascular smooth muscle cell‐derived matrix vesicles to enhance mineralization. Circ Res. 2011;109:e1‐e12. 21566214 10.1161/CIRCRESAHA.110.238808

[34] Dai XY , Zhao MM , Cai Y , et al. Phosphate‐induced autophagy counteracts vascular calcification by reducing matrix vesicle release. Kidney Int. 2013;83:1042‐1051. 23364520 10.1038/ki.2012.482

[35] Trajkovic K , Hsu C , Chiantia S , et al. Ceramide triggers budding of exosome vesicles into multivesicular endosomes. Science. 2008;319:1244‐1247. 18309083 10.1126/science.1153124

[36] Zeidan YH , Jenkins RW , Hannun YA . Remodeling of cellular cytoskeleton by the acid sphingomyelinase/ceramide pathway. J Cell Biol. 2008;181:335‐350. 18426979 10.1083/jcb.200705060 PMC2315679

[37] Kuang SQ , Kwartler CS , Byanova KL , et al. Rare, nonsynonymous variant in the smooth muscle‐specific isoform of myosin heavy chain, Myh 11, R247c, alters force generation in the aorta and phenotype of smooth muscle cells. Circ Res. 2012;110:1411‐1422. 22511748 10.1161/CIRCRESAHA.111.261743 PMC3917690

[38] Bobryshev YV , Killingsworth MC , Huynh TG , Lord RS , Grabs AJ , Valenzuela SM . Are calcifying matrix vesicles in atherosclerotic lesions of cellular origin? Basic Res Cardiol. 2007;102:133‐143. 17136418 10.1007/s00395-006-0637-9

[39] Chen YD , Fang YT , Cheng YL , et al. Exophagy of Annexin A2 via Rab11, Rab8a and Rab27a in Ifn‐gamma‐stimulated lung epithelial cells. Sci Rep. 2017;7:5676. 28720835 10.1038/s41598-017-06076-4 PMC5516008

[40] Cui L , Houston DA , Farquharson C , MacRae VE . Characterisation of matrix vesicles in skeletal and soft tissue mineralisation. Bone. 2016;87:147‐158. 27072517 10.1016/j.bone.2016.04.007

[41] Bardeesi ASA , Gao J , Zhang K , et al. A novel role of cellular interactions in vascular calcification. J Transl Med. 2017;15:95. 28464904 10.1186/s12967-017-1190-z PMC5414234

[42] Christianson HC , Svensson KJ , van Kuppevelt TH , Li JP , Belting M . Cancer cell exosomes depend on cell‐surface heparan sulfate proteoglycans for their internalization and functional activity. Proc Natl Acad Sci U S A. 2013;110:17380‐17385. 24101524 10.1073/pnas.1304266110 PMC3808637

[43] Shibata M , Shigematsu T , Hatamura I , et al. Reduced expression of perlecan in the aorta of secondary hyperparathyroidism model rats with medial calcification. Ren Fail. 2010;32:214‐223. 20199184 10.3109/08860220903367544

[44] Jiao X , Billings PC , O'Connell MP , Kaplan FS , Shore EM , Glaser DL . Heparan sulfate proteoglycans (Hspgs) modulate Bmp2 osteogenic bioactivity in C2c12 cells. J Biol Chem. 2007;282:1080‐1086. 17020882 10.1074/jbc.M513414200

[45] Yu X , Odenthal M , Fries JW . Exosomes as mirna carriers: formation‐function‐future. Int J Mol Sci. 2016;17:pii: E2028. 27918449 10.3390/ijms17122028 PMC5187828

[46] Cui Y , Luan J , Li H , Zhou X , Han J . Exosomes derived from mineralizing osteoblasts promote St2 cell osteogenic differentiation by alteration of microrna expression. FEBS Lett. 2016;590:185‐192. 26763102 10.1002/1873-3468.12024

[47] Chaturvedi P , Chen NX , O'Neill K , et al. Differential mirna expression in cells and matrix vesicles in vascular smooth muscle cells from rats with kidney disease. PLoS ONE. 2015;10:e0131589. 26115487 10.1371/journal.pone.0131589 PMC4482652

[48] Kubota S , Chiba M , Watanabe M , Sakamoto M , Watanabe N . Secretion of small/micrornas including Mir‐638 into extracellular spaces by sphingomyelin phosphodiesterase 3. Oncol Rep. 2015;33:67‐73. 25394686 10.3892/or.2014.3605 PMC4254672

[49] Feng W , Zhang K , Liu Y , et al. Apocynin attenuates angiotensin Ii‐induced vascular smooth muscle cells osteogenic switching via suppressing extracellular signal‐regulated kinase 1/2. Oncotarget. 2016;7:83588‐83600. 27835878 10.18632/oncotarget.13193 PMC5347790

[50] Ouyang L , Zhang K , Chen J , Wang J , Huang H . Roles of platelet‐derived growth factor in vascular calcification. J Cell Physiol. 2017;233:2804‐2814. 28467642 10.1002/jcp.25985

[51] Balderman JA , Lee HY , Mahoney CE , et al. Bone morphogenetic protein‐2 decreases microrna‐30b and microrna‐30c to promote vascular smooth muscle cell calcification. J Am Heart Assoc. 2012;1:e003905. 23316327 10.1161/JAHA.112.003905 PMC3540659

[52] Liu J , Xiao X , Shen Y , et al. Microrna‐32 promotes calcification in vascular smooth muscle cells: implications as a novel marker for coronary artery calcification. PLoS ONE. 2017;12:e0174138. 28319142 10.1371/journal.pone.0174138 PMC5358880

[53] Hao J , Zhang L , Cong G , Ren L , Hao L . Microrna‐34b/C inhibits aldosterone‐induced vascular smooth muscle cell calcification via a Satb2/Runx2 pathway. Cell Tissue Res. 2016;366:733‐746. 27503378 10.1007/s00441-016-2469-8

[54] Goettsch C , Rauner M , Pacyna N , Hempel U , Bornstein SR , Hofbauer LC . Mir‐125b regulates calcification of vascular smooth muscle cells. Am J Pathol. 2011;179:1594‐1600. 21806957 10.1016/j.ajpath.2011.06.016 PMC3181383

[55] Panizo S , Naves‐Diaz M , Carrillo‐Lopez N , et al. Micrornas 29b, 133b, and 211 regulate vascular smooth muscle calcification mediated by high phosphorus. J Am Soc Nephrol. 2016;27:824‐834. 26187577 10.1681/ASN.2014050520 PMC4769184

[56] Rangrez AY , M'Baya‐Moutoula E , Metzinger‐Le Meuth V , et al. Inorganic phosphate accelerates the migration of vascular smooth muscle cells: evidence for the involvement of Mir‐223. PLoS ONE. 2012;7:e47807. 23094093 10.1371/journal.pone.0047807 PMC3475714

[57] Cui RR , Li SJ , Liu LJ , et al. Microrna‐204 regulates vascular smooth muscle cell calcification in vitro and in vivo. Cardiovasc Res. 2012;96:320‐329. 22871591 10.1093/cvr/cvs258

[58] Chistiakov DA , Sobenin IA , Orekhov AN , Bobryshev YV . Human Mir‐221/222 in physiological and atherosclerotic vascular remodeling. Biomed Res Int. 2015;2015:354517. 26221589 10.1155/2015/354517 PMC4499635

[59] Mackenzie NC , Staines KA , Zhu D , Genever P , Macrae VE . Mirna‐221 and Mirna‐222 synergistically function to promote vascular calcification. Cell Biochem Funct. 2014;32:209‐216. 24604335 10.1002/cbf.3005 PMC4158883

[60] Gui T , Zhou G , Sun Y , et al. Micrornas that target Ca(2 + ) transporters are involved in vascular smooth muscle cell calcification. Lab Invest. 2012;92:1250‐1259. 22688076 10.1038/labinvest.2012.85

[61] Xia ZY , Hu Y , Xie PL , et al. Runx2/Mir‐3960/Mir‐2861 positive feedback loop is responsible for osteogenic transdifferentiation of vascular smooth muscle cells. Biomed Res Int. 2015;2015:624037. 26221600 10.1155/2015/624037 PMC4499372

[62] Xin H , Wang F , Li Y , et al. Secondary release of exosomes from astrocytes contributes to the increase in neural plasticity and improvement of functional recovery after stroke in rats treated with exosomes harvested from Microrna 133b‐Overexpressing multipotent mesenchymal stromal cells. Cell Transplant. 2017;26:243‐257. 27677799 10.3727/096368916X693031 PMC5303172

[63] Yentrapalli R , Merl‐Pham J , Azimzadeh O , et al. Quantitative changes in the protein and mirna cargo of plasma exosome‐like vesicles after exposure to ionizing radiation. Int J Radiat Biol. 2017;93:569‐580. 28264626 10.1080/09553002.2017.1294772

[64] Lunavat TR , Cheng L , Einarsdottir BO , et al. Brafv600 inhibition alters the microrna cargo in the vesicular secretome of malignant melanoma cells. Proc Natl Acad Sci U S A. 2017;114:E5930‐E5939. 28684402 10.1073/pnas.1705206114 PMC5530690

[65] Ulbing M , Kirsch AH , Leber B , et al. Micrornas 223‐3p and 93‐5p in patients with chronic kidney disease before and after renal transplantation. Bone. 2017;95:115‐123. 27866993 10.1016/j.bone.2016.11.016 PMC6326349

[66] Kroemer G , Marino G , Levine B . Autophagy and the integrated stress response. Mol Cell. 2010;40:280‐293. 20965422 10.1016/j.molcel.2010.09.023 PMC3127250

[67] Xu M , Liu L , Song C , Chen W , Gui S . Ghrelin improves vascular autophagy in rats with vascular calcification. Life Sci. 2017;179:23‐29. 27916732 10.1016/j.lfs.2016.11.025

[68] Mihaylova MM , Shaw RJ . The Ampk signalling pathway coordinates cell growth, Autophagy and Metabolism.. Nat Cell Biol. 2011;13:1016‐1023. 21892142 10.1038/ncb2329 PMC3249400

[69] Sudo R , Sato F , Azechi T , Wachi H . 7‐ketocholesterol‐induced lysosomal dysfunction exacerbates vascular smooth muscle cell calcification via oxidative stress. Genes Cells. 2015;20:982‐991. 26419830 10.1111/gtc.12301

[70] Blanc L , Vidal M . New insights into the function of rab gtpases in the context of exosomal secretion. Small GTPases. 2017;9:95‐106. 28135905 10.1080/21541248.2016.1264352 PMC5902209

[71] Savina A , Fader CM , Damiani MT , Colombo MI . Rab11 promotes docking and fusion of multivesicular bodies in a calcium‐dependent manner. Traffic. 2005;6:131‐143. 15634213 10.1111/j.1600-0854.2004.00257.x

[72] Villarroya‐Beltri C , Gutierrez‐Vazquez C , Sanchez‐Cabo F , et al. Sumoylated Hnrnpa2b1 controls the sorting of mirnas into exosomes through binding to specific motifs. Nat Commun. 2013;4:2980. 24356509 10.1038/ncomms3980 PMC3905700

[73] Mattoscio D , Casadio C , Miccolo C , et al. Autophagy regulates Ubc9 Levels during viral‐mediated tumorigenesis. PLoS Pathog. 2017;13:e1006262. 28253371 10.1371/journal.ppat.1006262 PMC5349695

[74] Ceneri N , Zhao L , Young BD , et al. Rac2 modulates atherosclerotic calcification by regulating macrophage interleukin‐1beta production. Arterioscler Thromb Vasc Biol. 2017;37:328‐340. 27834690 10.1161/ATVBAHA.116.308507 PMC5269510

[75] Henaut L , Sanchez‐Nino MD , Aldamiz‐Echevarria Castillo G , et al. Targeting local vascular and systemic consequences of inflammation on vascular and cardiac valve calcification. Expert Opin Ther Targets. 2016;20:89‐105. 26788590 10.1517/14728222.2015.1081685

[76] Ikeda K , Souma Y , Akakabe Y , et al. Macrophages play a unique role in the plaque calcification by enhancing the osteogenic signals exerted by vascular smooth muscle cells. Biochem Biophys Res Commun. 2012;425:39‐44. 22820183 10.1016/j.bbrc.2012.07.045

[77] Bretz NP , Ridinger J , Rupp AK , et al. Body fluid exosomes promote secretion of inflammatory cytokines in monocytic cells via Toll‐like receptor signaling. J Biol Chem. 2013;288:36691‐36702. 24225954 10.1074/jbc.M113.512806 PMC3868779

[78] Liao L , Zhou Q , Song Y , et al. Ceramide mediates Ox‐Ldl‐induced human vascular smooth muscle cell calcification Via P38 Mitogen‐activated protein kinase signaling. PLoS ONE. 2013;8:e82379. 24358176 10.1371/journal.pone.0082379 PMC3865066

[79] Bostrom K . Proinflammatory vascular calcification. Circ Res. 2005;96:1219‐1220. 15976320 10.1161/01.RES.0000172407.20974.e5

[80] Wang C , Zhang C , Liu L , et al. Macrophage‐derived Mir‐155‐containing exosomes suppress fibroblast proliferation and promote fibroblast inflammation during cardiac injury. Mol Ther. 2017;25:192‐204. 28129114 10.1016/j.ymthe.2016.09.001 PMC5363311

[81] Ismail N , Wang Y , Dakhlallah D , et al. Macrophage microvesicles induce macrophage differentiation and Mir‐223 transfer. Blood. 2013;121:984‐995. 23144169 10.1182/blood-2011-08-374793 PMC3567345

[82] Watson KE , Bostrom K , Ravindranath R , Lam T , Norton B , Demer LL . Tgf‐Beta 1 and 25‐hydroxycholesterol stimulate osteoblast‐like vascular cells to calcify. J Clin Invest. 1994;93:2106‐2113. 8182141 10.1172/JCI117205 PMC294336

[83] Wen X , Liu A , Yu C , et al. Inhibiting post‐translational core fucosylation prevents vascular calcification in the model of uremia. Int J Biochem Cell Biol. 2016;79:69‐79. 27521658 10.1016/j.biocel.2016.08.015

[84] Matsubara T , Tanaka N , Patterson AD , Cho JY , Krausz KW , Gonzalez FJ . Lithocholic acid disrupts phospholipid and sphingolipid homeostasis leading to cholestasis in mice. Hepatology. 2011;53:1282‐1293. 21480330 10.1002/hep.24193 PMC3077083

[85] Hu G , Yao H , Chaudhuri AD , et al. Exosome‐mediated shuttling of microrna‐29 regulates Hiv Tat and morphine‐mediated neuronal dysfunction. Cell Death Dis. 2012;3:e381. 22932723 10.1038/cddis.2012.114 PMC3434655

[86] Liang C , Bu S , Fan X . Suppressive effect of Microrna‐29b on hepatic stellate cell activation and its crosstalk with Tgf‐Beta1/Smad3. Cell Biochem Funct. 2016;34:326‐333. 27273381 10.1002/cbf.3193 PMC5089641

[87] Byon CH , Javed A , Dai Q , et al. Oxidative stress induces vascular calcification through modulation of the osteogenic transcription factor Runx2 by Akt signaling. J Biol Chem. 2008;283:15319‐15327. 18378684 10.1074/jbc.M800021200 PMC2397455

[88] Wei Q , Ren X , Jiang Y , Jin H , Liu N , Li J . Advanced glycation end products accelerate Rat vascular calcification through rage/oxidative stress. BMC Cardiovasc Disord. 2013;13:13. 23497312 10.1186/1471-2261-13-13 PMC3626911

[89] Kay AM , Simpson CL , Stewart JA Jr . The role of age/rage signaling in diabetes‐mediated vascular calcification. J Diabetes Res. 2016;2016:6809703. 27547766 10.1155/2016/6809703 PMC4980539

[90] Patel GK , Khan MA , Bhardwaj A , et al. Exosomes confer chemoresistance to pancreatic cancer cells by promoting ros detoxification and Mir‐155‐mediated suppression of key gemcitabine‐metabolising enzyme, Dck. Br J Cancer. 2017;116:609‐619. 28152544 10.1038/bjc.2017.18 PMC5344296

[91] Hagiwara S , McClelland A , Kantharidis P . Microrna in diabetic nephropathy: renin angiotensin, age/rage, and oxidative stress pathway. J Diabetes Res. 2013;2013:173783. 24575418 10.1155/2013/173783 PMC3875101

[92] Gong M , Yu B , Wang J , et al. Mesenchymal stem cells release exosomes that transfer mirnas to endothelial cells and promote angiogenesis. Oncotarget. 2017;8:45200‐45212. 28423355 10.18632/oncotarget.16778 PMC5542178

[93] Liu H , Wang J , Chen Y , et al. Npc‐Exs alleviate endothelial oxidative stress and dysfunction through the Mir‐210 downstream Nox2 and Vegfr2 pathways. Oxid Med Cell Longev. 2017;2017:9397631. 28630660 10.1155/2017/9397631 PMC5467331

[94] Hansson GK , Libby P , Schonbeck U , Yan ZQ . Innate and adaptive immunity in the pathogenesis of atherosclerosis. Circ Res. 2002;91:281‐291. 12193460 10.1161/01.res.0000029784.15893.10

[95] Chen D , Huang X , Yang M , Gan H , Gunawan EJ , Tang W . Treg/Th17 functional disequilibrium in chinese uremia on hemodialysis: a link between calcification and cardiovascular disease. Ren Fail. 2012;34:697‐702. 22503035 10.3109/0886022X.2012.672155

[96] Danyan C , Xiaolong H , Song L , Hua G , Weixue T , Ke L . The effects of Rhbmp‐2 and Treg/Th17 functional disequilibrium in uremic patients with cardiovascular complication after maintenance hemodialysis. Int J Artif Organs. 2013;36:464‐472. 23897228 10.5301/ijao.5000217

[97] Li P , Liu C , Yu Z , Wu M . New insights into regulatory T cells: exosome‐ and non‐coding Rna‐mediated regulation of homeostasis and resident treg cells. Front Immunol. 2016;7:574. 27999575 10.3389/fimmu.2016.00574 PMC5138199

[98] Bordon Y , Regulatory T . Cells: a message of peace. Nat Rev Immunol. 2014;14:581. 25086648 10.1038/nri3729

[99] Okoye IS , Coomes SM , Pelly VS , et al. Microrna‐containing T‐regulatory‐cell‐derived exosomes suppress pathogenic T helper 1 cells. Immunity. 2014;41:89‐103. 25035954 10.1016/j.immuni.2014.05.019 PMC4104030

[100] Balachandran K , Sucosky P , Jo H , Yoganathan AP . Elevated cyclic stretch induces aortic valve calcification in a bone morphogenic protein‐dependent manner. Am J Pathol. 2010;177:49‐57. 20489151 10.2353/ajpath.2010.090631 PMC2893650

[101] Pironti G , Strachan RT , Abraham D , et al. Circulating exosomes induced by cardiac pressure overload contain functional angiotensin Ii type 1 receptors. Circulation. 2015;131:2120‐2130. 25995315 10.1161/CIRCULATIONAHA.115.015687 PMC4470842

[102] Csiszar A , Labinskyy N , Smith KE , et al. Downregulation of bone morphogenetic protein 4 expression in coronary arterial endothelial cells: role of shear stress and the camp/protein kinase a pathway. Arterioscler Thromb Vasc Biol. 2007;27:776‐782. 17272757 10.1161/01.ATV.0000259355.77388.13

[103] Jae N , McEwan DG , Manavski Y , Boon RA , Dimmeler S . Rab7a and Rab27b control secretion of endothelial microrna through extracellular vesicles. FEBS Lett. 2015;589:3182‐3188. 26348397 10.1016/j.febslet.2015.08.040

